# Combination of a third generation bisphosphonate and replication-competent adenoviruses augments the cytotoxicity on mesothelioma

**DOI:** 10.1186/s12885-016-2483-y

**Published:** 2016-07-12

**Authors:** Yuanyuan Jiang, Boya Zhong, Kiyoko Kawamura, Takao Morinaga, Masato Shingyoji, Ikuo Sekine, Yuji Tada, Koichiro Tatsumi, Hideaki Shimada, Kenzo Hiroshima, Masatoshi Tagawa

**Affiliations:** Division of Pathology and Cell Therapy, Chiba Cancer Center Research Institute, 666-2 Nitona, Chuo-ku, Chiba 260-8717 Japan; Department of Molecular Biology and Oncology, Graduate School of Medicine, Chiba University, Chiba, Japan; Division of Respirology, Chiba Cancer Center, Chiba, Japan; Department of Medical Oncology, Faculty of Medicine, University of Tsukuba, Tsukuba, Japan; Department of Respirology, Graduate School of Medicine, Chiba University, Chiba, Japan; Department of Surgery, School of Medicine, Toho University, Tokyo, Japan; Department of Pathology, Tokyo Women’s Medical University Yachiyo Medical Center, Yachiyo, Japan

**Keywords:** Mesothelioma, Replication-competent adenovirus, Bisphosphonates, p53

## Abstract

**Background:**

Approximately 80 % of mesothelioma specimens have the wild-type *p53* gene, whereas they contain homozygous deletions in the INK4A/ARF locus that encodes *p14*^*ARF*^ and the *16*^*INK4A*^ genes. Consequently, the majority of mesothelioma is defective of the p53 pathways. We examined whether zoledronic acid (ZOL), a third generation bisphosphonate, and adenoviruses with a deletion of the *E1B-55kD* gene (Ad-delE1B55), which augments p53 levels in the infected tumors, could produce combinatory anti-tumor effects on human mesothelioma cells bearing the wild-type *p53* gene.

**Methods:**

Cytotoxicity of ZOL and Ad-delE1B55 was assessed with a WST assay. Cell cycle changes were tested with flow cytometry. Expression levels of relevant molecules were examined with western blot analysis to investigate a possible mechanism of cytotoxicity. Furthermore, the expressions of Ad receptors on target cells and infectivity were estimated with flow cytometry. Viral replication was assayed with the tissue culture infection dose method.

**Results:**

A combinatory use of ZOL and Ad-delE1B55 suppressed cell growth and increased sub-G1 or S-phase populations compared with a single agent, depending on cells tested. The combinatory treatment up-regulated p53 levels and subsequently enhanced the cleavage of caspase-3, 8, 9 and poly (ADP-ribose) polymerase, but expression of molecules involved in autophagy pathways were inconsistent. ZOL-treated cells also increased Ad infectivity with a dose-dependent manner and augmented Ad replication although the expression levels of integrin molecules, one of the Ad receptors, were down-regulated.

**Conclusions:**

These findings indicated that ZOL and Ad-delE1B55 achieved combinatory anti-tumor effects through augmented apoptotic pathways or increased viral replication.

**Electronic supplementary material:**

The online version of this article (doi:10.1186/s12885-016-2483-y) contains supplementary material, which is available to authorized users.

## Background

Malignant pleural mesothelioma, developed in the pleural cavity, is often associated with asbestos exposure in the patient history [1, 2]. The prognosis remains poor and mesothelioma is resistant to a number of chemotherapeutic and molecular-targeting agents. A novel therapeutic strategy is thereby required to improve the prognosis. Mesothelioma has a characteristic genetic change. Previous analyses showed that approximately 80 % of mesothelioma specimens had a homozygous deletion in the INK4A/ARF locus which encoded *p14*^*ARF*^ and the *16*^*INK4A*^ genes, but the *p53* genotype was infrequently mutated [3, 4]. The genetic defect leads to inactivation of the p53 pathways and may be linked with decreased susceptibility to anti-cancer agents.

Bisphosphonates are synthetic analogues of pyrophosphates and show high binding affinity to mineralized bone matrix [5]. Previous reports showed that bisphosphonates produced cytotoxic effects on tumors such as breast and prostate cancer [6, 7], and these cytotoxic actions were attributable to a number of mechanisms including apoptosis induction and anti-angiogenesis [5, 8]. Zoledronic acid (ZOL), one of the third generation of bisphosphonates, inhibits the farnesyl pyrophosphate synthetase, a key enzyme in the mevalonate pathway, and depletes isoprenoid pools, which subsequently results in decreased prenylation of small guanine-nucleotide-binding regulatory proteins (small G proteins) [5]. Consequently, ZOL prevented growth, adhesion or spreading, and invasion of cancer cells [5, 9]. In our previous study, we demonstrated ZOL-mediated cytotoxic effects on mesothelioma cells [10] and showed that ZOL treatments improved cytotoxicity of adenoviruses (Ad) expressing the *p53* gene on mesothelioma [11]. Further analyses indicated that augmentation of p53 by ZOL was essential in combinatory effects of ZOL and DNA damaging drugs which included the first-line anti-cancer agents for mesothelioma [11].

Replicating-competent Ad is a new strategy for cancer therapy. They can spread and destroy tumors without deleterious effects in normal tissues [12, 13]. The replicable Ad continuously release the progenies from infected tumors and consequently circumvent low transduction efficacy. This replicable propensity enhances the cytotoxicity but host immunity can be inhibitory to the viral spreading. Ad lacking the E1B-55 kDa molecules (Ad-delE1B55) are replication-competent and were originally hypothesized to target only *p53*-mutated or -null tumors due to the defect in p53-inactivating E1B-55 kDa protein [14]. Nevertheless, Ad proteins that are synthesized during the replication also regulate p53 expression in infected cells at various levels even in an epigenetic manner [15]. Subsequent studies in fact showed that Ad-delE1B55-induced cytotoxicity was not always related to the *p53* genotype [16]. Moreover, our previous study showed that Ad-delE1B55 produced cytotoxicity on mesothelioma cells with the wild-type *p53* gene and achieved combinatory anti-tumor agents with the first-line chemotherapeutic agents [17].

In the present study, we examined whether ZOL and Ad-delE1B55 could produce combinatory anti-tumor effects on human mesothelioma cells carrying the wild-type *p53* gene. We speculated that both agents augmented endogenous p53 levels, which resulted in augmentation of the cytotoxicity. Furthermore, we analyzed a possible mechanism of the combination and investigated involvement of apoptotic pathways and viral replication in the anti-tumor effects.

## Methods

### Cells

Human mesothelioma cells, MSTO-211H, NCI-H28, NCI-H226, NCI-H2452 cells, all of which were purchased from American Type Culture Collection (Manassas, VA, USA), and EHMES-10 (provided from Dr. Hironobu Hamada, Hiroshima University, Japan) [18] and were cultured with RPMI 1640 medium with 10 % fetal calf serum. HEK 293 and A549 cells, derived from American Type Culture Collection and Dr. Katsuyuki Hamada (Ehime University), respectively, were cultured with in Dulbecco’s Modified Eagle’s Medium containing 10 % fetal calf serum. NCI-H28, NCI-H2452 and EHMES-10 cells are defective of the *p14*^*ARF*^ and *p16*^*INK4A*^ genes, and MSTO-211H and NCI-H226 cells lack the *p14* and *p16* transcription (Additional file 1: Figure S1). Sequence analyses showed that all of them possessed the wild-type *p53* gene.

### Ad preparation

Replication-competent Ad-delE1B55 (Accession number for Ad; M73260), in which the 55 kDa molecule-encoding E1B region (corresponding to 2019–3509 in M73260 sequences) is deleted, and replication-incompetent Ad expressing the *ß-galactosidase* (NM066611) (Ad-LacZ) or the g*reen fluorescent protein* gene (U55762) (Ad-GFP) powered by the cytomegalovirus promoter (KU317610), were prepared with an Adeno-X expression system (Takara, Shiga, Japan) and HEK293 cells. The numbers of virus particles (vp) per ml was estimated with the formula [absorbance at 260 nm of purified Ad in the presence of 0.1 % sodium dodecyl sulfate].

### Cell cycle analysis and Giemsa staining

Cells treated with ZOL (Novartis, Basel, Switzerland) and/or either Ad-delE1B55 or Ad-LacZ as a control were fixed in ice-cold 100 % ethanol, incubated with RNase (50 μg/ml) and stained with propidium iodide (50 μg/ml). The staining profiles were analyzed with FACSCalibur and CellQuest software (BD Biosciences, CA, USA). We set up a gated area for data collection to remove doublets signals (Additional file 2: Figure S2). For Giemsa staining, cells treated with ZOL and/or Ad-delE1B55 were treated with colcemid (10 μg/ml). They were further incubated with hypotonic buffer and stained with Giemsa solution.

### Viability test in vitro

Cells (5 × 10^3^/well) in 96-well plates were cultured with ZOL and/or Ad-delE1B55 for 5 days. Cell viability was determined with a cell-counting WST kit (Wako, Osaka, Japan) and the relative viability was calculated based on the absorbance without any treatments. Viable cell numbers were also counted with the trypan blue dye exclusion test. Combinatory effects were examined with CalcuSyn software (Biosoft, Cambridge, UK). Combination index (CI) values at respective fractions affected (Fa) points, which showed relative suppression levels of the cell viability, were calculated based on the WST assay. CI < 1, CI = 1 and CI > 1 indicate synergistic, additive and antagonistic actions, respectively.

### Western blot analysis

Cells were cultured with ZOL and/or either Ad-delE1B55 or Ad-LacZ, and the cell lysate was subjected to sodium dodecyl sulfate polyacrylamide gel electrophoresis. The protein was transferred to a nylon filter and was hybridized with antibodies (Ab), against p53 (Thermo Fisher Scientific, Fremont, CA, USA), phosphorylated p53 at serine (Ser) 15, Bid (detecting truncated-Bid as well), caspase-3, cleaved caspase-3, caspase-8, cleaved caspase-8, caspase-9, cleaved caspase-9, poly (ADP-ribose) polymerase (PARP), Beclin-1, Atg5, LC3A/B (Cell Signaling, Danvers, MA, USA), cyclin A, cyclin E, type 2/5 Ad E1A (Santa Cruz Biotech, Dallas, TX, USA), phosphorylated H2 histone family member (H2AX) at Ser 139 (Biolegend, San Diego, CA, USA), hexon (Abcam, Cambridge, UK) and α-Tubulin (Thermo Fisher Scientific) as a loading control. The membranes were developed with the ECL system (GE Healthcare, Buckinghamshire, UK).

### Infectivity with Ad-GFP and expression of Ad receptors

Cells were infected with Ad-GFP at several vp doses for 30 min and were then washed to remove Ad. Infected cells were cultured for 48 h and then analyzed for percentages of GFP-positive cells with FACSCalibur and CellQuest software. Cells of which fluorescence was greater than the brightest 5 % of uninfected cells were judged as positively stained. For detecting Ad receptors, cells were stained with anti-coxsackie adenovirus receptors (CAR) (Upstate, Charlottesville, VA, USA), integrin αvβ3 (Chemicon, Billerica, MA, USA), integrin αvβ5 (Abcam) Ab and the fluorescence intensity was analyzed with FACSCalibur and CellQuest software.

### Virus production

Cells infected with Ad-delE1B55 were treated with or without ZOL. The cell lysates were examined for the cytotoxicity with A549 cells and the virus titers were calculated with the median tissue culture infectious dose (TCID_50_) method.

## Results

### Cytotoxic activities of ZOL and Ad-delE1B55 on mesothelioma

**Fig. 1:**
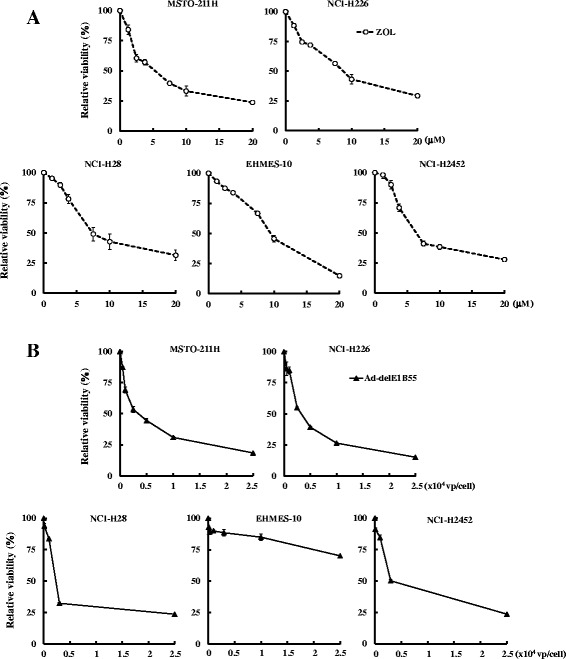
A growth inhibitory activity of ZOL or Ad-delE1B55 on mesothelioma.** a** Cells were treated with various concentrations of ZOL for 5 days and the cell viabilities were measured with the WST assay. The relative viabilities were calculated based on the absorbance without ZOL treatments. Means of triplicated samples and SE bars are shown (*n* = 3). **b** Cells were infected with various amounts of Ad-delE1B55 and the viability was tested with the WST assay 5 days after the infection. Relative viability was calculated based on uninfected cells. Averages and SE bars are shown (*n* = 3). We repeated this assay for 3 times and show representative data

We investigated possible cytotoxic effects produced by ZOL or Ad-delE1B55 on 5 kinds of mesothelioma, MSTO-211H, NCI-H226, NCI-H28, EHMES-10 and NCI-H2452 cells, which are defective of the *p14*^*ARF*^ and *p16*^*INK4A*^ genes or the transcription but possess the wild-type *p53*. Cells treated with ZOL showed decreased viability with a similar level (Fig. 1a), whereas the sensitivity to Ad-delE1B55 was different among the cells and EHMES-10 cells were resistant (Fig. 1b). Moreover, a separate experiment and a previous study showed that p53 of NCI-H2452 cells was truncated [19]. We therefore focused on MSTO-211H, NCI-H226 and NCI-H28 cells for further analyses, which were sensitive to both agents.

**Fig. 2:**
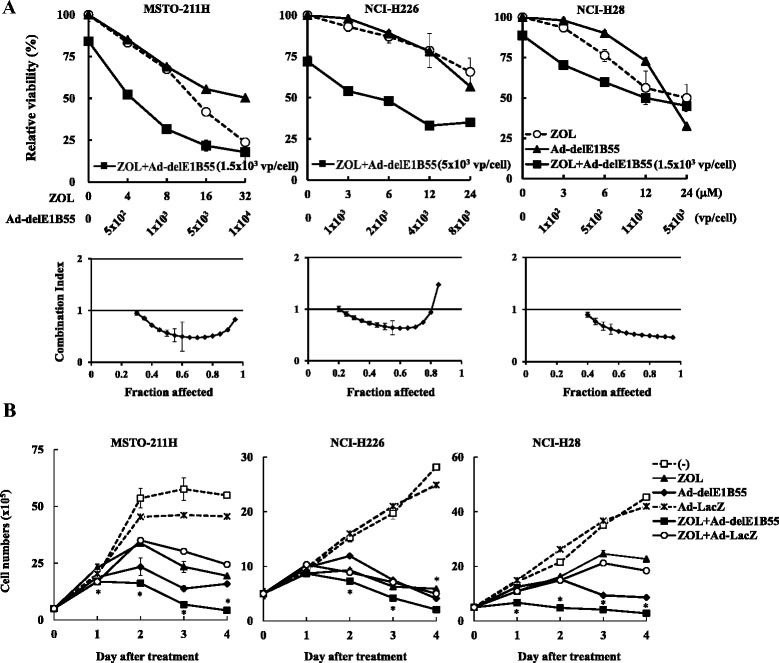
Combinatory effects produced by ZOL and Ad-delE1B55. **a** Cells were treated with different concentrations of ZOL and doses of Ad-delE1B55 as indicated for 5 days. As for combination of ZOL and Ad-delE1B55, cells were treated with different concentrations of ZOL and Ad-delE1B at a fixed dose (MSTO-211H and NCI-H28: 1.5 × 10^3^ vp/cell, NCI-H226: 5 × 10^3^ vp/cell). The cell viabilities were measured with the WST assay. Averages and SE bars are shown (*n* = 3). CI values based on the cell viabilities were determined by different Fa points with the CalcuSyn software. We repeated this assay for 3 times and show representative data. **b** Proliferation of cells treated with ZOL and/or either Ad-delE1B55 or Ad-LacZ as a control. Cells were treated with ZOL (MSTO-211H: 10 μM, NCI-H226: 60 μM, NCI-H28: 100 μM) and/or Ad-delE1B55 or Ad-LacZ (MSTO-211H: 4.5 × 10^3^ vp/cell, NCI-H226: 2 × 10^3^ vp/cell, NCI-H28: 3 × 10^3^ vp/cell). Live cell numbers were determined with the dye exclusion test. Averages and SEs are shown. We repeated this assay for 3 times in total and show representative data. *p < 0.05, compared the ZOL plus Ad-delE1B55-treated group with Ad-LacZ-, ZOL-, Ad-delE1B55-, ZOL plus Ad-LacZ-treated cells groups

We next examined cytotoxicity produced by a combinatory use of ZOL and Ad-delE1B55 (Fig. 2a). The combination achieved cytotoxicity greater than a single treatment. We examined the combinatory effects with the CalcuSyn software and showed that CI values were below 1 at most of the Fa points tested (between 0.3 and 0.8 in MSTO-211H and NCI-H226 cells and between 0.4 and 0.9 in NCI-H28 cells). These data indicated that the combination of ZOL and Ad-delE1B55 achieved additive or synergistic cytotoxicity in most of the cases. We noticed that NCI-H226 and NCI-H28 but not MSTO-211H cells were relatively resistant to ZOL-mediated cell death assayed with the dye exclusion test although these cells were similarly sensitive to ZOL in the WST assay. The discrepancy was derived from differential measuring systems between the WST assay, detecting production of mitochondria-derived energy, and the dye exclusion test, detecting membrane permeability. We then treated NCI-H226 and NCI-H28 cells with relatively high ZOL concentrations (60–100 μM) to induce the growth inhibition and cell death in the following experiments. In contrast, the high ZOL concentrations completely killed MSTO-211H cells and we treated the cells at 10 μM. We then examined live cell numbers assayed with the dye exclusion test (Fig. 2b). Cell growth was gradually retarded and declined depending on the agents and cells, and the growth inhibition was stronger in the combination than in cells treated with ZOL or Ad-delE1B55 alone. Replication-incompetent Ad-LacZ as a control minimally suppressed the proliferation and did not produce combinatory effects with ZOL. These data collectively indicated that ZOL and Ad-delE1B55 achieved combinatory anti-tumor effects on mesothelioma.

### Cell cycle changes induced by ZOL and Ad-delE1B55

**Fig. 3:**
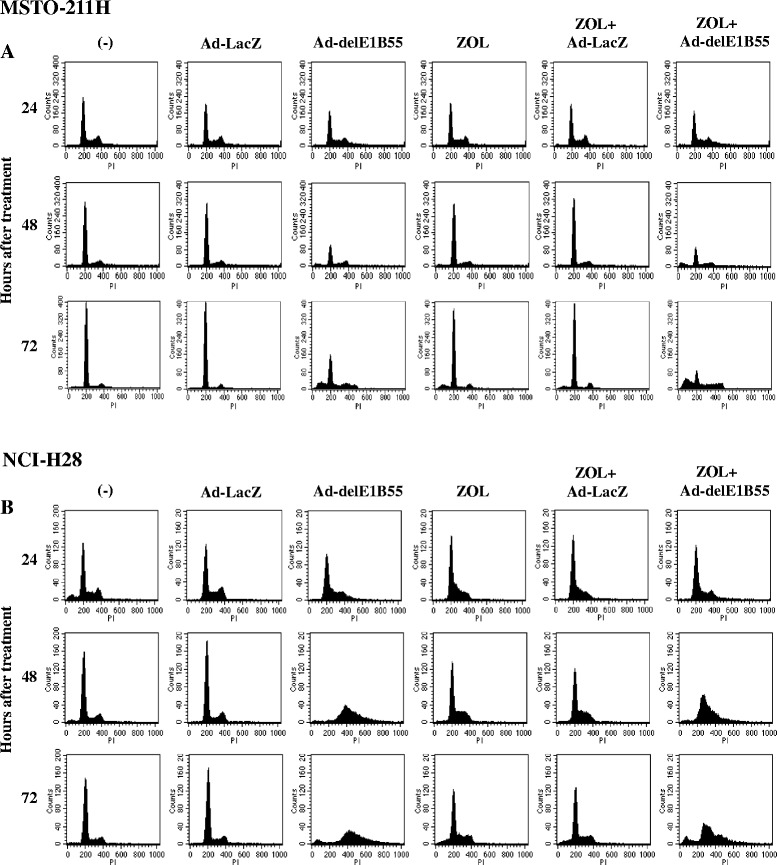
Representative profiles of cell-cycle progression. MSTO-211H (**a**) or NCI-H28 cells (**b**) were treated with ZOL (MSTO-211H: 10 μM; NCI-H28: 80 μM) and/or either Ad-delE1B55 or Ad-LacZ (MSTO-211H: 4.5 × 10^3^ vp/cell; NCI-H226: 2 × 10^3^ vp/cell). The cell cycle progression was analyzed with flow cytometry at 24, 48 and 72 h after the treatments

We examined cell cycle changes in MSTO-211H and NCI-H28 cells with flow cytometry (Fig. 3, Tables 1 and 2) since NCI-H226 cells constantly showed relative high sub-G1 fractions. Ad-delE1B55 treatments increased hyperploid fractions, more than 4 N populations, and sub-G1 fractions in MSTO-211H cells, whereas ZOL increased sub-G1 fractions (Fig. 3a, Table 1). A combinatory use of ZOL and Ad-delE1B55 further increased sub-G1 populations in MSTO-211H cells compared with treatments of Ad-delE1B55 or ZOL alone, indicating that combination induced further apoptosis. NCI-H28 cells infected with Ad-delE1B55 showed increased G2/M phase populations and hyperploid fractions greater than Ad-delE1B55-infected MSTO-211H cells, and ZOL treatments in NCI-H28 cells augmented S-phase populations with minimal induction of sub-G1 fractions (Fig. 3b, Table 2). Combination of both agents in NCI-H28 cells increased S-phase and greater than treatments with a single agent, and minimally up-regulated sub-G1 populations. The increased S-phase fraction in NCI-H28 cells may be attributable to failure of Ad-delE1B55-infected cells with the hyperploid fraction to enter into mitosis and consequently the cell cycle were arrested in S-phase and/or G2/M-phase. We also confirmed the increased S-phase and G2/M-phase in NCI-H28 cells treated with ZOL and Ad-delE1B55 with western blot analysis (Additional file 3: Figure S3). These cells showed decreased cyclin E and enhanced cyclin A expression levels, which was compatible with cells in S-phase entry and in a progress from S- to G2/M-phase. Cells uninfected or infected with Ad-LacZ as a control however showed a minimal level of polyploidy and the sub-G1 fractions did not increase markedly in both MSTO-211H and NCI-H28 cells with Ad-LacZ.

**Table 1:** Cell cycle progression of MSTO-211H cells treated with ZOL and/or Ad-delE1B55

Time (hrs)	Treatment	Cell cycle distribution (% ± SE)				
		Sub-G1	G1	S	G2/M	>4 N
24	(−)	2.04 ± 0.04	58.80 ± 0.20	18.17 ± 0.24	20.67 ± 0.31	0.81 ± 0.06
	Ad-LacZ	2.90 ± 0.16	57.30 ± 0.19	17.59 ± 0.22	20.97 ± 0.26	1.83 ± 0.04
	Ad-delE1B55	2.61 ± 0.16	55.29 ± 0.07	20.41 ± 0.11	20.08 ± 0.27	2.22 ± 0.11
	ZOL	2.47 ± 0.05	57.47 ± 0.10	18.34 ± 0.27	20.70 ± 0.22	1.62 ± 0.09
	ZOL + Ad-LacZ	3.58 ± 0.17	55.98 ± 0.16	17.94 ± 0.17	20.84 ± 0.10	2.22 ± 0.08
	ZOL + Ad-delE1B55	3.72 ± 0.05	53.99 ± 0.11	20.53 ± 0.23	19.42 ± 0.35	3.00 ± 0.09
48	(−)	1.73 ± 0.07	76.54 ± 0.12	8.20 ± 0.03	12.59 ± 0.08	1.18 ± 0.04
	Ad-LacZ	1.37 ± 0.08	75.90 ± 0.38	8.60 ± 0.16	12.55 ± 0.33	1.82 ± 0.03
	Ad-delE1B55	12.25 ± 0.04	51.87 ± 0.21	12.47 ± 0.13	21.56 ± 0.19	2.32 ± 0.03
	ZOL	3.82 ± 0.08	76.77 ± 0.43	6.70 ± 0.09	11.31 ± 0.33	1.60 ± 0.01
	ZOL + Ad-LacZ	2.81 ± 0.11	76.08 ± 0.33	7.47 ± 0.12	11.95 ± 0.13	1.96 ± 0.06
	ZOL + Ad-delE1B55	19.69 ± 0.18^a^	47.47 ± 0.19^a^	15.42 ± 0.44^a^	15.23 ± 0.35	2.69 ± 0.16
72	(−)	0.96 ± 0.02	87.55 ± 0.01	3.77 ± 0.13	7.26 ± 0.19	0.59 ± 0.05
	Ad-LacZ	1.46 ± 0.10	87.58 ± 0.30	2.75 ± 0.05	7.79 ± 0.14	0.51 ± 0.03
	Ad-delE1B55	22.52 ± 0.07	43.47 ± 0.18	11.57 ± 0.14	12.89 ± 0.23	10.14 ± 0.23
	ZOL	11.19 ± 0.08	76.77 ± 0.28	3.45 ± 0.08	7.93 ± 0.20	0.81 ± 0.12
	ZOL + Ad-LacZ	6.44 ± 0.03	80.27 ± 0.15	3.04 ± 0.04	9.66 ± 0.13	0.78 ± 0.04
	ZOL + Ad-delE1B55	35.52 ± 0.05^a^	24.06 ± 0.30^a^	13.89 ± 0.13^b^	11.83 ± 0.21	15.41 ± 0.19
96	(−)	1.03 ± 0.07	87.25 ± 0.01	4.43 ± 0.03	6.93 ± 0.06	0.50 ± 0.03
	Ad-LacZ	1.16 ± 0.06	87.36 ± 0.28	3.34 ± 0.17	7.65 ± 0.11	0.68 ± 0.04
	Ad-delE1B55	12.14 ± 0.27	53.92 ± 0.29	11.93 ± 0.06	13.21 ± 0.27	9.32 ± 0.31
	ZOL	9.32 ± 0.11	80.72 ± 0.21	3.98 ± 0.06	5.73 ± 0.07	0.40 ± 0.07
	ZOL + Ad-LacZ	8.93 ± 0.17	78.40 ± 0.17	4.22 ± 0.14	7.92 ± 0.13	0.79 ± 0.04
	ZOL+ Ad-delE1B55	40.75 ± 0.08^a^	21.50 ± 0.21^a^	14.26 ± 0.11^b^	10.58 ± 0.12	13.59 ± 0.18^b^

Cells were treated with ZOL (10 μM) and/or either Ad-delE1B55 or Ad-LacZ (4.5 × 10^3^ vp/cell) and cultured for 24–96 h. Cell cycle profiles were analyzed with flow cytometry. Averages with SEs are shown (*n* = 3)

^a^ *p* < 0.01, ^b^ *p* < 0.05; compared between ZOL + Ad-delE1B55-treated cells, and Ad-LacZ, ZOL-, Ad-delE1B55-, ZOL + Ad-LacZ-treated cells

**Table 2:** Cell cycle progression of NCI-H28 cells treated with ZOL and/or Ad-delE1B55

Time (hrs)	Treatment	Cell cycle distribution (% ± SE)				
		Sub-G1	G1	S	G2/M	>4 N
24	(−)	8.57 ± 0.34	57.61 ± 1.07	15.48 ± 0.53	16.96 ± 0.89	0.92 ± 0.21
	Ad-LacZ	0.17 ± 0.02	58.59 ± 0.71	18.33 ± 0.19	22.24 ± 0.39	1.18 ± 0.17
	Ad-delE1B55	0.25 ± 0.10	59.35 ± 0.68	20.28 ± 0.08	15.33 ± 0.38	5.45 ± 0.19
	ZOL	0.16 ± 0.03	61.76 ± 0.64	25.71 ± 0.31	212.15 ± 0.48	0.89 ± 0.05
	ZOL + Ad-LacZ	0.15 ± 0.03	67.62 ± 0.48	21.85 ± 0.35	9.87 ± 0.20	0.96 ± 0.07
	ZOL + Ad-delE1B55	0.23 ± 0.03	65.45 ± 0.73	17.33 ± 0.21	14.21 ± 0.38	3.24 ± 0.24
48	(−)	2.79 ± 0.15	75.8 ± 0.13	9.30 ± 0.05	13.66 ± 0.20	0.93 ± 0.06
	Ad-LacZ	0.24 ± 0.01	73.42 ± 0.15	9.22 ± 0.21	16.30 ± 0.20	1.08 ± 0.13
	Ad-delE1B55	1.17 ± 0.05	1.70 ± 0.22	8.77 ± 0.22	36.84 ± 0.12	51.80 ± 0.28
	ZOL	0.86 ± 0.10	58.00 ± 0.54	25.64 ± 0.15	15.28 ± 0.27	0.82 ± 0.11
	ZOL + Ad-LacZ	0.70 ± 0.03	60.80 ± 0.20	25.40 ± 0.27	12.92 ± 0.28	0.77 ± 0.03
	ZOL + Ad-delE1B55	1.44 ± 0.22	19.93 ± 1.20	47.98 ± 1.01^a^	1.28 ± 0.32	0.64 ± 0.37
72	(−)	1.06 ± 0.02	76.33 ± 0.64	9.26 ± 0.23	12.68 ± 0.28	0.92 ± 0.21
	Ad-LacZ	0.51 ± 0.06	76.20 ± 0.37	8.68 ± 0.26	13.50 ± 0.46	1.41 ± 0.07
	Ad-delE1B55	6.67 ± 0.17	2.15 ± 0.23	5.12 ± 0.49	27.45 ± 0.26	59.21 ± 0.85
	ZOL	5.67 ± 0.23	59.46 ± 0.21	19.55 ± 0.57	14.86 ± 0.47	1.02 ± 0.07
	ZOL + Ad-LacZ	6.07 ± 0.13	61.94 ± 0.30	16.93 ± 0.16	14.56 ± 0.12	0.97 ± 0.11
	ZOL + Ad-delE1B55	8.56 ± 0.38^a^	7.77 ± 0.84	41.33 ± 1.20^a^	22.49 ± 0.42	20.77 ± 0.11

Cells were treated with ZOL (80 μM) and/or either Ad-delE1B55 or Ad-LacZ (2 × 10^3^ vp/cell) and cultured for 24–72 h. Cell cycle profiles were analyzed with flow cytometry. Averages with SEs are shown (*n* = 3)

^a^ *p* < 0.01; compared between ZOL + Ad-delE1B55-treated cells, and Ad-LacZ-, ZOL-, Ad-delE1B55-, ZOL + Ad-LacZ-treated cells

**Fig. 4:**
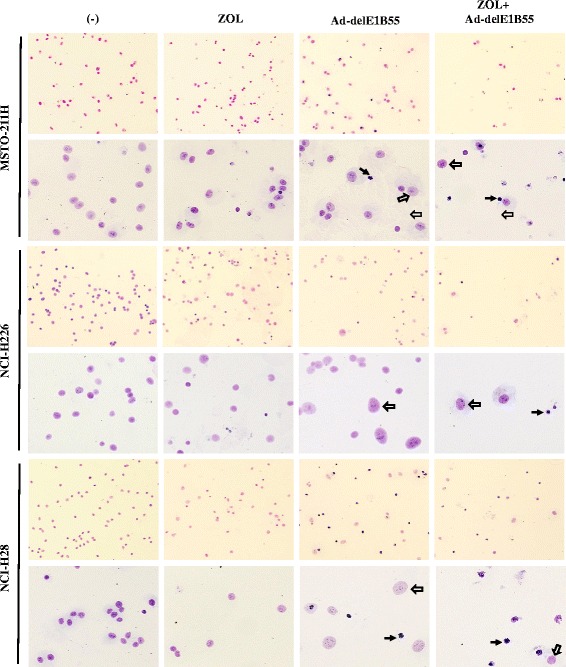
Giemsa staining after Ad-delE1B55 infection. Cells were treated with ZOL (MSTO-211H: 10 μM, NCI-H226: 60 μM, NCI-H28: 80 μM) and/or Ad-delE1B55 (MSTO-211H: 2 × 10^3^ vp/cell, NCI-H226: 1 × 10^3^ vp/cell, NCI-H28: 2 × 10^3^ vp/cell) for 72 h. Nuclear morphological changes were examined after the Giemsa staining. In Ad-delE1B55-treated cells, small and highly condensed nucleus (solid arrow) and large and uncondensed nucleus (open arrow) were detected. An upper panel and lower panel are x4 and x10 in magnifications, respectively

We also examined nuclear configurations of MSTO-211H, NCI-H226 and NCI-H28 cells with Giemsa staining (Fig. 4). These cells treated with ZOL did not show any changed compared untreated cells. In contrast, Ad-delE1B55 infections increased cells with enlarged and condensed nuclei, which suggested increased nuclear DNA contents and pyknotic changes. Combinatory treatments also showed the same nuclear changes with decreased cell numbers, indicating that cell proliferation was inhibited by the treatments.

### Molecular changes induced by combination of ZOL and Ad-delE1B55

**Fig. 5:**
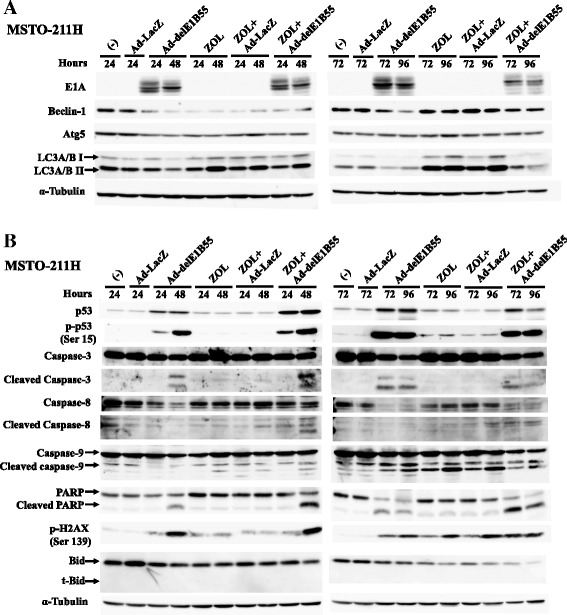
Expression of molecules linked with cell death. MSTO-211H cells were treated with ZOL (20 μM) and Ad-delE1B55 or Ad-LacZ (3 × 10^3^ vp/cell) as a control. Cells were cultured for the indicated times. Expression of viral proteins and autophagy pathways (**a**) and molecules linked with cell death pathways (**b**) were examined with respective Ab and α-Tubulin was used as a loading control

We examined a possible mechanism of combinatory effects produced by ZOL and Ad-delE1B55 in MSTO-211H cells with western blot analyses (Fig. 5). E1A became detectable at 24 h after the infection, but the expression levels were not different from those with additional 10 μM of ZOL (Fig. 5a). We also examined a possible involvement of autophagy pathways. Beclin-1 expression levels were down-regulated in cells treated with either Ad-delE1B55 or ZOL at 24 and 48 h, and in Ad-delE1B55-treated cells at 72 and 96 h. Atg5 expression levels in cells treated with Ad-delE1B55 or ZOL minimally decreased at 24 and 48 h but unchanged after 72 h. Both Beclin-1 and Atg5 expression levels in the combination-treated cells were not different from those in cells treated with a single agent except Beclin-1 expression in Ad-delE1B55-treated cells at 96 h. Moreover, there was no major transition from LC3A/B I to LC3A/B II accompanied by any treatments including the combination although the expression levels of LC3A/B II and to less extent LC3A/B I were enhanced in ZOL-treated cells. In addition, the LC3A/B II expression levels in the combination corresponded to the levels of between Ad-delE1B55-treated and ZOL-treated cells. Expression levels of Beclin-1, Atg5 and LC3A/B were therefore differentially influenced by the agents. These data collectively indicated that cytotoxicity induced by ZOL might be partly linked with autophagy, but Ad-delE1B- and the combination-induced cell death were irrelevant to autophagy.

We then examined expression levels of apoptosis-linked proteins (Fig. 5b). ZOL at 10 μM in MSTO-211H cells did not influence p53 or phosphorylated p53 levels, or induce cleavages of caspase-3, −8 and PARP, but minimally augmented that of caspase-9. In contrast, Ad-delE1B55-treated cells augmented p53 and phosphorylated p53 levels, and cleavages of caspase-3,-9 and PARP, but not that of caspase-8. Apoptosis by ZOL or Ad-delE1B55 could thus be differentially activated through intrinsic or extrinsic pathways. Cells treated with the combination showed up-regulated p53 expression, p53 phosphorylation, and subsequently enhanced cleavage of caspase-3, 8, 9 and PARP at a greater level than those treated with ZOL or Ad-delE1B alone at 24 and 48 h. The up-regulated expression in the combination was however not detected after 72 h except the cleavage PARP. We noticed that Bid expression was down-regulated in the combination at 96 h, but truncated Bid, which contributes to the linkage between the death receptor- and the mitochondria-mediated apoptosis, was not induced. Interestingly, phosphorylated H2AX molecules, a marker of DNA damages, was augmented in Ad-delE1B55- and ZOL-treated cells, suggesting that DNA damages were involved in both type of the cell death. The combination did not increase the phosphorylated H2AX levels greater than the summated levels of Ad-delE1B- and ZOL-treated cases. These data collectively indicated that the combinatory cytotoxicity at an early phase up to 48 h was at least partly due to augmented p53 levels and activated downstream apoptosis, but activation of the p53 downstream pathways was less significantly attributable to the cytotoxicity at the later phase.

### Expression of Ad receptors and Ad infectivity after ZOL treatments

**Fig. 6:**
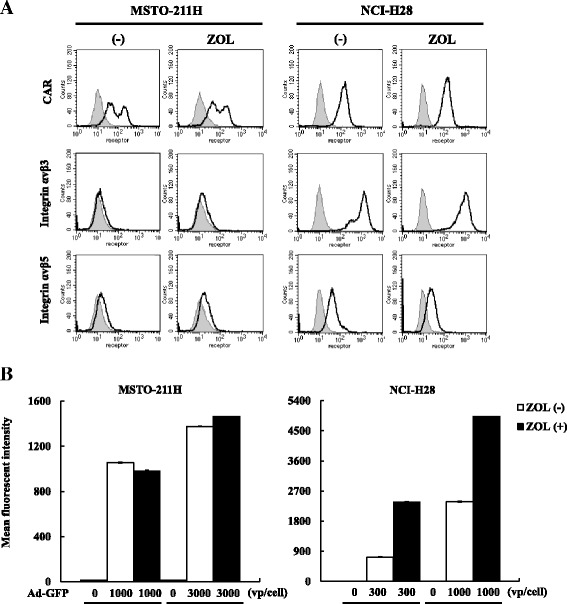
Expression of Ad receptors and infectivity of Ad. **a** Expression levels of CAR and integrin αvβ3 or αvβ5 molecules on MSTO-211H and NCI-H28 cells were analyzed with flow cytometry. Representative profiles of cells untreated or treated with ZOL (MSTO-211H: 10 μM, NCI-H28: 80 μM) for 48 h are shown. CAR expression on MSTO-211H cells was always biphasic and both low and high CAR-positive cells were detected Shaded profiles are those stained with 2nd Ab alone. **b** Efficacy of Ad infection examined with Ad-GFP. Cells were infected with Ad-GFP (300–3,000 vp/cell) and treated with or without ZOL (MSTO-211H: 10 μM, NCI-H28: 80 μM) for 48 h. The mean fluorescent intensity of the GFP-positive cells was analyzed with flow cytometry and expressed with an arbitrary unit. Averages and the SE bars are shown (*n* = 3)

We examined changes of CAR and integrin αvβ3 or αvβ5 expression levels by ZOL treatments on MSTO-211H and NCI-H28 cells (Fig. 6a, Table 3). Influence of ZOL on the expression level, which was shown as mean fluorescent intensity, was calculated by comparing the level of ZOL-treated cells and that of untreated cells as a control (Table 3). ZOL (10 μM) scarcely influenced expression levels on MSTO-211H cells, whereas ZOL (80 μM) decreased the levels of integrin αvβ3 and αvβ5 on NCI-H28 cell. We also examined effects of ZOL at different concentrations with NCI-H28 cells and found that increased doses of ZOL decreased the expression levels of integrin in particular αvβ5 (Additional file 4: Table S1). We could not examine ZOL-mediated effects on MSTO-211H cells at the high concentrations since they became dead at these doses.

**Table 3:** Expression of Ad receptor molecules after ZOL treatments

Cells	Ab	ZOL treatment	Mean fluorescent intensity (average ± SE)	% radio (average ± SE)
MSTO-211H	2nd Ab alone	(−)	12.42 ± 0.07	
		(+)	13.44 ± 0.07	108.19 ± 0.51
	CAR	(−)	95.21 ± 2.04	
		(+)	95.86 ± 0.09	100.69 ± 0.09
	Integrin αvβ3	(−)	13.59 ± 0.02	
		(+)	14.67 ± 0.04	107.92 ± 0.04
	Integrin αvβ5	(−)	15.64 ± 0.10	
		(+)	18.12 ± 0.03	115.88 ± 0.64
NCI-H28	2nd Ab alone	(−)	11.39 ± 0.03	
		(+)	11.55 ± 0.86	101.38 ± 0.79
	CAR	(−)	127.26 ± 0.31	
		(+)	119.29 ± 0.69	93.73 ± 0.03
	Integrin αvβ3	(−)	1006.61 ± 1.24	
		(+)	718.29 ± 2.33	71.36 ± 0.12
	Integrin αvβ5	(−)	44.01 ± 0.09	
		(+)	22.01 ± 0.03	50.01 ± 0.13

Cells untreated or treated with ZOL (MSTO-211H: 10 μM, NCI-H28: 80 μM) for 48 h were subjected to flow cytometry to detect expression of the cellular receptors of type 5 Ad. Means fluorescence intensity was expressed as an arbitrary unit and influence of ZOL on the expression levels was expressed as a percent ratio based on respective ZOL-untreated cases as a control (*n* = 3)

We then examined Ad infectivity on mesothelioma using Ad-GFP, replication-incompetent type 5 Ad with the same receptor usage as Ad-delE1B55 (Fig. 6b). Fluorescence intensity of GFP on MSTO-211H cells was not significantly different after ZOL treatments (10 μM) but that on NCI-H28 cells increased with ZOL (80 μM). We also tested the GFP intensity with different ZOL concentrations, and the infectivity to NCI-H28 cells increased in a dose-dependent manner (Additional file 5: Figure S4). These results indicated that ZOL influenced Ad receptor expression and infectivity of Ad-delE1B55, but these Ad receptor expression levels did not directly correlated with Ad infectivity. Nevertheless, the enhanced infectivity suggested a certain role in the combinatory cytotoxicity in NCI-H28 cells.

### Effects of ZOL on viral proliferations of Ad-delE1B55

**Fig. 7:**
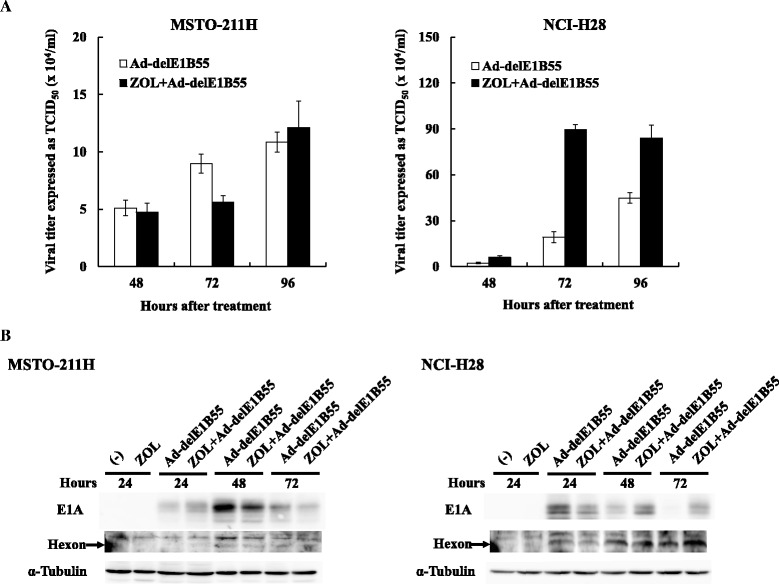
Influence of ZOL on viral progeny production. MSTO-211H and NCI-H28 cells were treated with Ad-delE1B55 (MSTO-211H: 4.5 × 10^3^ vp/cell, NCI-H28: 2 × 10^3^ vp/cell) or in combination of ZOL (MSTO-211H: 10 μM, NCI-H28: 80 μM) and the cell lysate was extracted at the indicated times. **a** The viral titers were assayed with the TCID_50_ method. Averages and SE bars are shown (*n* = 3). **b** Expression of E1A and hexon in cell lysate was probed with respective Ab. An upper band detected with anti-hexon Ab was due to a non-specific reaction and the lower band corresponded to hexon. α-Tubulin is used as a loading control

We examined whether the combinatory effects were associated with increased production of the viral progenies. MSTO-211H and NCI-H28 cells were infected with Ad-delE1B55 and treated with ZOL, and then the cell lysate was tested for the viral titers with the TCID_50_ method using A549 cells (Fig. 7a). The viral production in MSTO-211H cells remained unchanged except ZOL-treated cells at 72 h which decreased the production. In contrast, ZOL treatments in NCI-H28 cells augmented the viral propagations. Enhanced cytotoxicity by the combination in MSTO-211H cells were thereby irrelevant to production of infectious Ad progenies, whereas that in NCI-H28 cells could be attributable to the enhanced production of viral progenies. We also examined expression of E1A, the early gene product, and hexon, one of the late gene product after ZOL treatments (Fig. 7b). E1A expression in MSTO-211H cells remained unchanged or decreased with ZOL treatments, and hexon expression was not markedly different between ZOL-untreated and ZOL-treated cells. In contrast, E1A expression in NCI-H28 cells was initially lower in ZOL-treated cells at 24 h than in ZOL-untreated cells, but became greater in ZOL-treated than ZOL-untreated cells at 48 and 72 h. Hexon expression likewise was up-regulated by ZOL treatments after 48 h. The E1A and the hexon expression levels in NCI-H28 cells were correlated with production of viral progenies.

## Discussion

In this study, we demonstrated that ZOL and Ad-delE1B55 induced growth suppression and the combination of both agents produced additive or synergistic inhibitory effects on mesothelioma cells. ZOL and Ad-delE1B55 induced differential cell cycle changes and the combinatory effects were achieved through enhanced apoptosis or increased viral replication. It is the first report to our knowledge that bisphosphonates and replication-competent Ad produced combinatory effects.

We noticed that MSTO-211H cells were sensitive to ZOL with the dye exclusion test, but NCI-H226 and NCI-H28 cells required a relatively high dose to induce cell growth suppression although these 3 kinds of cells were similarly susceptible to ZOL with the WST assay. We then tested MSTO-211H cells with a low ZOL concentration and NCI-H226 and NCI-H28 cells with a high ZOL doses in other experimental conditions. Cell cycle analyses showed that Ad-delE1B55 and ZOL produced differential effects which was characterized by hyperploidy and S-phase arrest. A precise mechanism of hyperploidy and S-phase arrest remain currently unknown, but the present study showed that Ad-infected cells induced enlarged nuclei followed by pyknotic configurations, which was compatible with hyperploidy and apoptotic cell death. In addition, augmented cyclin A and decreased cyclin E expression suggested induction of S-phase arrest, and phosphorylation of H2AX indicated activation of a cellular system detecting viral DNA increase. Hyperploidy could be due to a direct or an indirect consequence of accumulated viral DNA and an activated DNA damage sensor system thereafter. Furthermore, increased S-phase populations could be resulted from impaired cell cycle progression at S- and G2-phase and from failure of cells to shift into mitosis. Previous studies in fact reported that ZOL induced S-phase arrest and that the cell cycle changes were subjected to aberrant signals induced by mutated tumor suppressor genes [20].

The majority of human mesothelioma possesses the wild type *p53* gene but lacks the *p14*^*ARF*^ and the *16*^*INK4A*^ genes, which subsequently leads to loss of the p53 functions and activation of the pRb pathways, respectively. ZOL activated endogenous p53 downstream pathways on mesothelioma even though the cell death did not depend on the p53 pathways in our recent study [10]. Nevertheless, up-regulated p53 levels increased sensitivity to cisplatin, one of the first-line chemotherapeutic agents for mesothelioma [11]. Consequently, ZOL facilitated DNA damage responses through the activated p53 downstream pathways despite p53 independence of ZOL-mediated cytotoxicity. On the other hand, Ad-delE1B55 also augmented endogenous p53 levels and subsequently activated the p53 functions in *p53* wild-type mesothelioma, which was evidenced by p53 phosphorylation, pRb dephosphorylation and cleavage of caspases [17]. The present study showed that combination of ZOL and Ad-delE1B55 increased phosphorylated p53 and cleaved caspase-3 and we therefore presume it rational to use the agent together from the viewpoint of activation of the p53 pathways. Moreover, effects of ZOL on Ad replication also needs to be clarified since viral replication itself induced cytotoxicity to the infected cells.

The ZOL inhibits prenylation of small G proteins and consequently suppresses functions of small G proteins [5, 8–10]. On the other hand, mesothelioma is often defective of the Hippo pathways due to mutation of the *NF*/*Merlin* gene [21, 22]. Loss of NF/Merlin functions leads to dysregulation of multiple signal pathways, and up-regulated functions of small G proteins is one of the uncontrolled regulation. Ras and RhoA family proteins, one of the major small G proteins, are in fact activated in malignant mesothelioma and subsequently mitogen-activated protein kinases are also activated in most of the mesothelioma cells [10, 23]. ZOL thereby blocks one of the dysfunctional Hippo pathways and combinatory effects of ZOL and Ad-delE1B55 can inhibit a possible cross-talk between NF/Merlin and the p53 pathways in mesothelioma with deletion of both INK4A/ARF and NF/Merlin regions.

We examined influence of ZOL on receptor expression levels and infectivity of Ad. Expression levels of major Ad receptors, CAR and integrin αvβ3 and αvβ5, were not affected by ZOL on MSTO-211H cells, whereas those of integrin but not CAR was down-regulated in NCI-H28 cells after ZOL treatments. The differential influence on integrin expression between the tested cells were not due to ZOL concentrations since ZOL at 10 μM also decreased integrin levels on NCI-H28 cells. Interestingly, Ad infectivity in NCI-H28 cells was rather enhanced after ZOL treatments despite the down-regulated integrin expression, indicating that the integrin as well as CAR did not play a crucial role in the infection in NCI-H28 cells. ZOL treatments induced alteration of actin fiber structures followed by morphological changes since small G proteins in particular Rho family proteins played a role in the organization of actin fibers in mesothelioma [24]. Moreover, a previous study reported that ZOL specifically suppressed integrin αvβ3 but not α5β1 expression through down-regulate focal adhesion kinase [25]. On the other hand, suppressed αvβ5 expression, demonstrated in the present study, had not yet been reported. Down-regulated expression of integrin molecules and enhanced Ad infectivity can be in part attributable to the ZOL-mediated morphological changes, but inhibition of small G proteins also influences several intracellular signal pathways and may augment infectivity, for example, due to facilitated viral release into cytoplasm after binding to the cellular receptors. Ad endocytosis via integrin molecules in fact requires activation of the lipid kinase phosphatidylinositol-3-OH kinase, which in turn augments signaling cascades of both Ras and Rho families [26]. In addition, RhoA, Cdc42 and Rab6 were targets for ZOL-induced actions including cell death, cell cycle arrest and modification of actin fiber structure [24]. These reports collectively suggested that combinatory cytotoxicity of ZOL and Ad-delE1B55 was linked with suppressed small G proteins functions. We also presume that increase infectivity could directly contribute to the enhanced production of Ad progenies since ZOL-treated MSTO-211H cells did not show the increased production but ZOL treatments augmented the production in NCI-211H cells. In addition, cells infected with Ad-delE1B55 during the G1 phase of cell cycle exhibited a reduced rate of viral late protein synthesis, and produced fewer viral progenies than cells infected during the S-phase [27]. Cell cycle arrest at S-phase that was observed in ZOL-treated NCI-H28 cells can therefore be favorable for enhanced viral progeny production.

We hypothesized a possible mechanism involved in the combinatory effects. ZOL increased an expression level of p53 in MSTO-211H cells, which was induced by Ad-delE1B55 at 48 h after the infection, and subsequently activated apoptotic signal pathways which was evidenced by up-regulated cleavages of caspase-8, −9 and −3, and PARP even without Bid truncation. Moreover, the combination markedly increased sub-G1 populations in MSTO-211H cells but ZOL did not influence progeny production of Ad-delE1B55. These data indicated that the combinatory effects in MSTO-211H cells were attributable to ZOL-mediated augmented p53 downstream pathways. In contrast, ZOL in NCI-H28 cells augmented the viral infection and the replication, which was also evidenced by elevated or prolonged expression of E1A and subsequent hexon expression. A possible mechanism of the up-regulated Ad infection can be linked with inhibited functions of small G proteins, and the augmented infection was dependent on cells. We noticed ZOL-mediated actions were different among mesothelioma [10, 20] and the present study showed a differential pattern of cell cycle progression and varied expression levels of cell surface molecules between MSTO-211H and NCI-28 cells. These differences are probably resulted from divergent effects of small G proteins in cellular functions among the cells tested.

Ad-delE1B55 has been approved in China as an anti-cancer agent for head and neck cancer [28] and ZOL is used in a clinical setting in many countries. We has also started a phase I clinical trial with an intrapleural injection of ZOL for mesothelioma patients, which can maintains a local concentration of ZOL to achieve cytotoxicity [29]. The present results under these circumstances will be one of the preclinical or relevant studies for a future clinical trial with replication-competent Ad and an inhibitor for small G proteins.

## Conclusions

In this study, we demonstrated that ZOL and Ad-delE1B55 induced growth arrest and produced combinatory cytotoxic effects on *p53* wild-type mesothelioma. We also showed that the combination of ZOL and Ad-delE1B55 produced anti-tumor effects through enhanced apoptotic pathways and up-regulated viral replication. These data collectively suggest a possible cross-talk of small G proteins and Ad replication-induced cell death in mesothelioma.

## Abbreviations

Ab, antibody; Ad, adenoviruses; Ad-delE1B55, Ad lacking the E1B-55 kDa molecules; CAR, coxsackie adenovirus receptor; CI, combination index; Fa, fractions affected; GFP, green fluorescent protein; H2AX, H2A histone family member X; LacZ, ß-galactosidase; PARP, poly (ADP-ribose) polymerase; small G proteins, small guanine-nucleotide-binding regulatory proteins; TCID_50_, median tissue culture infectious dose; vp, virus particles; ZOL, zoledronic acid

## Additional files

Additional file 1: Figure S1. Genetic deletion or loss of expression of the *p14* and *p16* genes in mesothelioma. (A) Polymerase chain reactions to detect the INK4A/ARF region, encoding the *p14* and *p16* genes. The *p14* gene is comprised by exon 1β, 2 and 3, and the *p16* is exon 1α, 2 and 3. NCI-H28, EHMES-10 and NCI-H2452 cells were defective of the *p14* and *p16* genes. (B) Reverse transcription-polymerase chain reactions to detect the *p14* and the *p16* transcripts with primers that amplified between exon 1β and 2 (for the *p14*) and exon 1α and 2 (for the *p16*). Met-5A cells and GAPDH (glyceraldehyde-3-phosphate dehydrogenase) were used for a positive and a loading control, respectively. MSTO-211H and NCI-H226 cells were negative for the *p14* and the *p16* transcripts. (PPTX 46 kb) Additional file 2: Figure S2. Gate control for data collection to remove doublet signals in flow cytometry**.** Cell cycle profiles of untreated cells were collected with flow cytometry and we set up a gate to collect the data in FL2-width (FL2-W) and FL2-area (FL-2A) (shown in a rectangle) and removed cell doublets in the collection processes. (PPTX 70 kb) Additional file 3: Figure S3. Expression of cyclin E and cyclin A. NCI-H28 cells treated with ZOL (80 μM) and/or either Ad-delE1B55 or Ad-LacZ (2 × 10^3^ vp/cell) for 48 h were subjected to western blot analysis. Cyclin E and A expression were probed with respective Ab and α-Tubulin was used as a loading control. (PPTX 84 kb) Additional file 4: Table S1. Expression Ad receptor molecules after ZOL treatments on NCI-H28 cells. (DOCX 21 kb) Additional file 5: Figure S4. Influence of ZOL on Ad infectivity. NCI-H28 cells were infected with Ad-GFP (300 or 1,000 vp/cell) and were treated with ZOL as indicated for 48 h. The mean fluorescent intensity of the GFP-positive cells was analyzed with flow cytometry and expressed with an arbitrary unit. Averages and the SE bars are shown (*n* = 3). Data of ZOL at 80 μM are the same as those in Fig. 6b. (PPTX 46 kb)
