# Supplementary material for: Combination of a third generation bisphosphonate and replication-competent adenoviruses augments the cytotoxicity on mesothelioma
Source: BMC Cancer. 2016 Jul 12;16:455. doi: 10.1186/s12885-016-2483-y (PMC4942884; doi:10.1186/s12885-016-2483-y)
Supplement: Additional file 1: Figure S1. — Genetic deletion or loss of expression of the p14 and p16 genes in mesothelioma. (A) Polymerase chain reactions to detect the INK4A/ARF region, encoding the p14 and p16 genes. The p14 gene is comprised by exon 1β, 2 and 3, and the p16 is exon 1α, 2 and 3. NCI-H28, EHMES-10 and NCI-H2452 cells were defective of the p14 and p16 genes. (B) Reverse transcription-polymerase chain reactions to detect the p14 and the p16 transcripts with primers that amplified between exon 1β and 2 (for the p14) and exon 1α and 2 (for the p16). Met-5A cells and GAPDH (glyceraldehyde-3-phosphate dehydrogenase) were used for a positive and a loading control, respectively. MSTO-211H and NCI-H226 cells were negative for the p14 and the p16 transcripts. (PPTX 46 kb) [file 12885_2016_2483_MOESM1_ESM.pptx]

## Slide 1
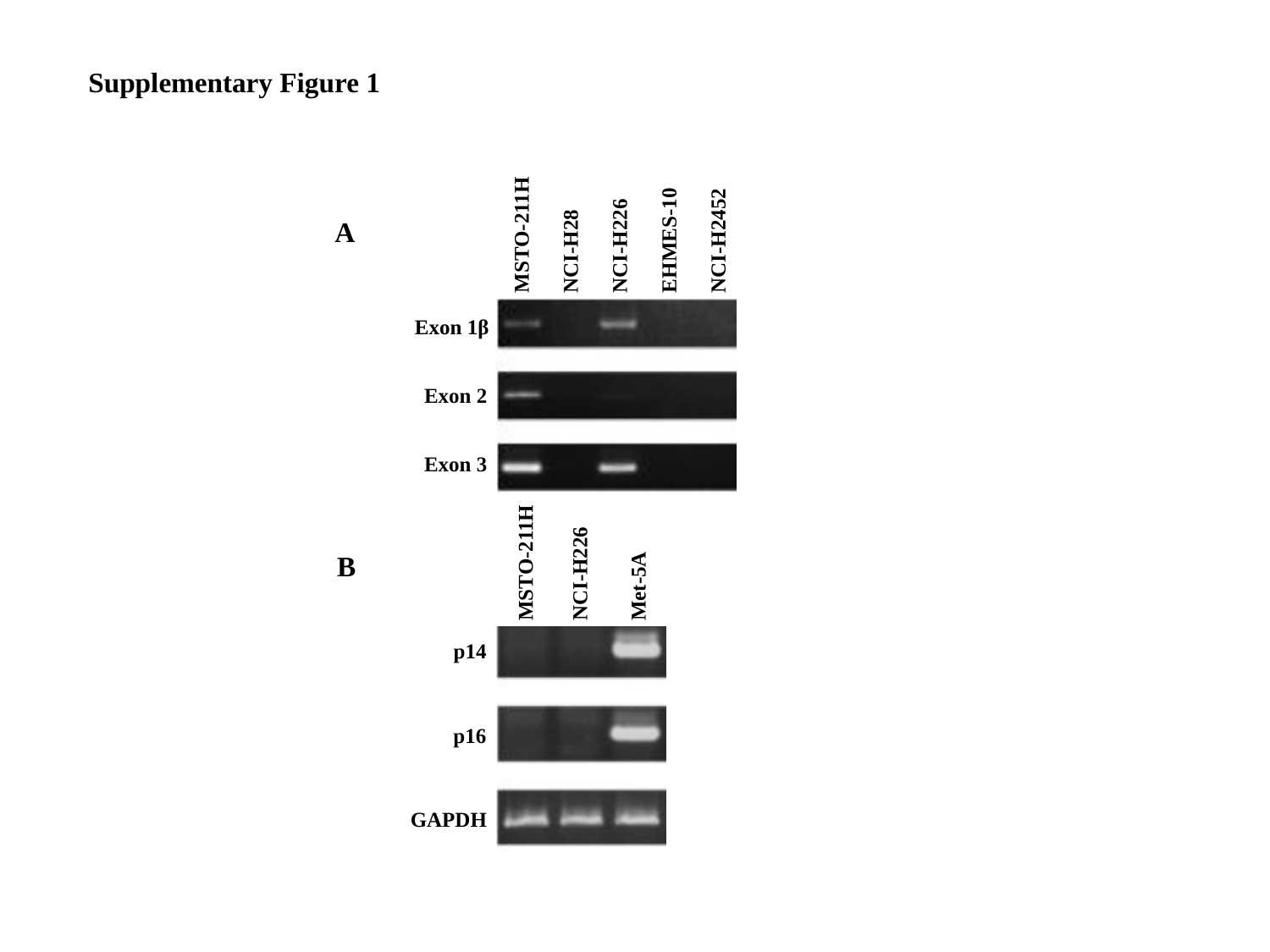

Supplementary Figure 1
A
MSTO-211H
NCI-H28
NCI-H226
EHMES-10
NCI-H2452
Exon 1β
Exon 2
Exon 3
MSTO-211H
NCI-H226
Met-5A
B
p14
p16
GAPDH
