# Supplementary figures and images for: Combination of a third generation bisphosphonate and replication-competent adenoviruses augments the cytotoxicity on mesothelioma
Source: BMC Cancer. 2016 Jul 12;16:455. doi: 10.1186/s12885-016-2483-y (PMC4942884; doi:10.1186/s12885-016-2483-y)

## Slide 1
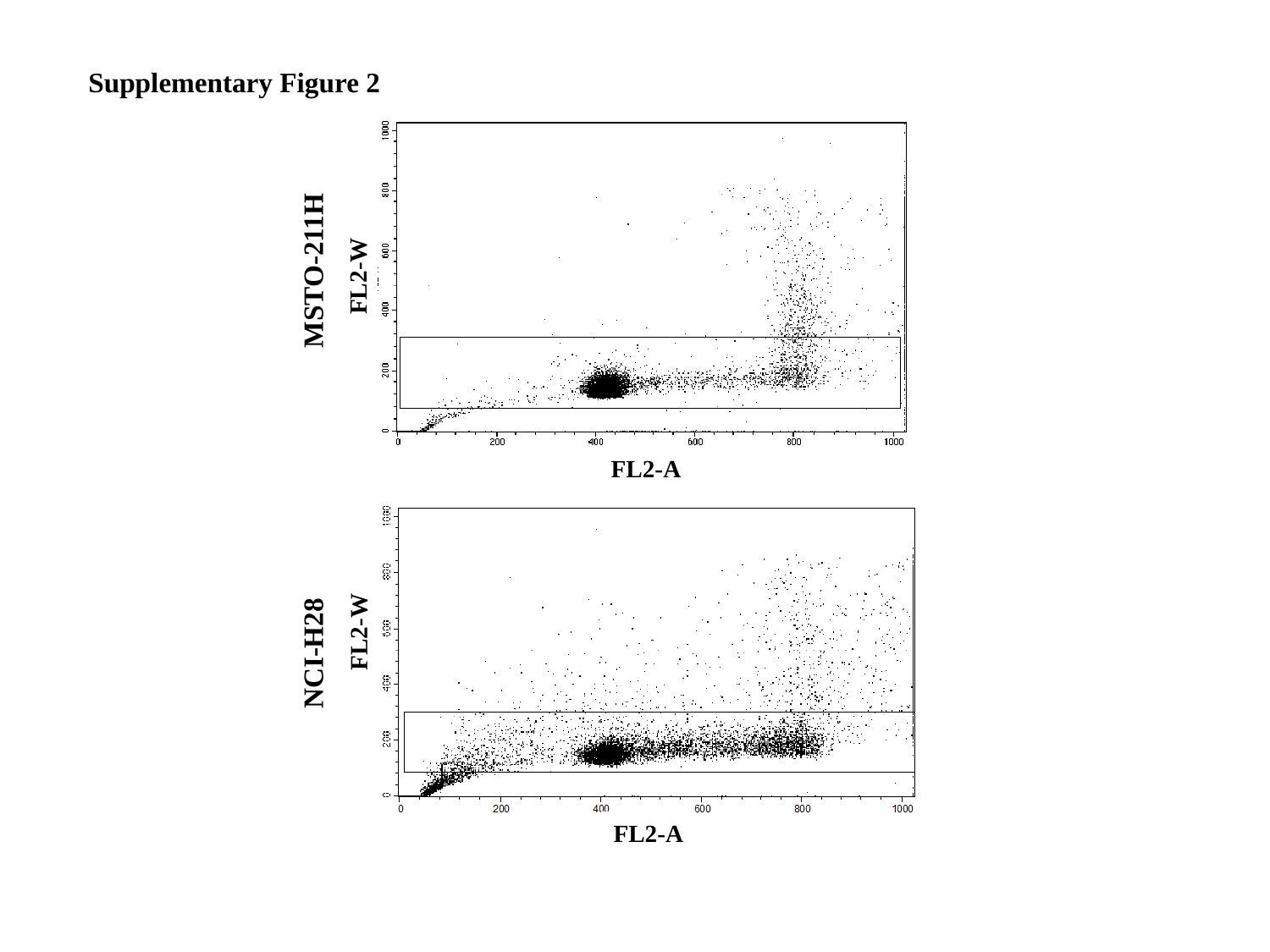

Supplementary Figure 2
MSTO-211H
FL2-W
FL2-A
FL2-W
NCI-H28
FL2-A

Supplement: Additional file 2: Figure S2. — Gate control for data collection to remove doublet signals in flow cytometry. Cell cycle profiles of untreated cells were collected with flow cytometry and we set up a gate to collect the data in FL2-width (FL2-W) and FL2-area (FL-2A) (shown in a rectangle) and removed cell doublets in the collection processes. (PPTX 70 kb) [file 12885_2016_2483_MOESM2_ESM.pptx]
