# Supplementary material for: Combination of a third generation bisphosphonate and replication-competent adenoviruses augments the cytotoxicity on mesothelioma
Source: BMC Cancer. 2016 Jul 12;16:455. doi: 10.1186/s12885-016-2483-y (PMC4942884; doi:10.1186/s12885-016-2483-y)
Supplement: Additional file 3: Figure S3. — Expression of cyclin E and cyclin A. NCI-H28 cells treated with ZOL (80 μM) and/or either Ad-delE1B55 or Ad-LacZ (2 × 10^3^ vp/cell) for 48 h were subjected to western blot analysis. Cyclin E and A expression were probed with respective Ab and α-Tubulin was used as a loading control. (PPTX 84 kb) [file 12885_2016_2483_MOESM3_ESM.pptx]

## Slide 1
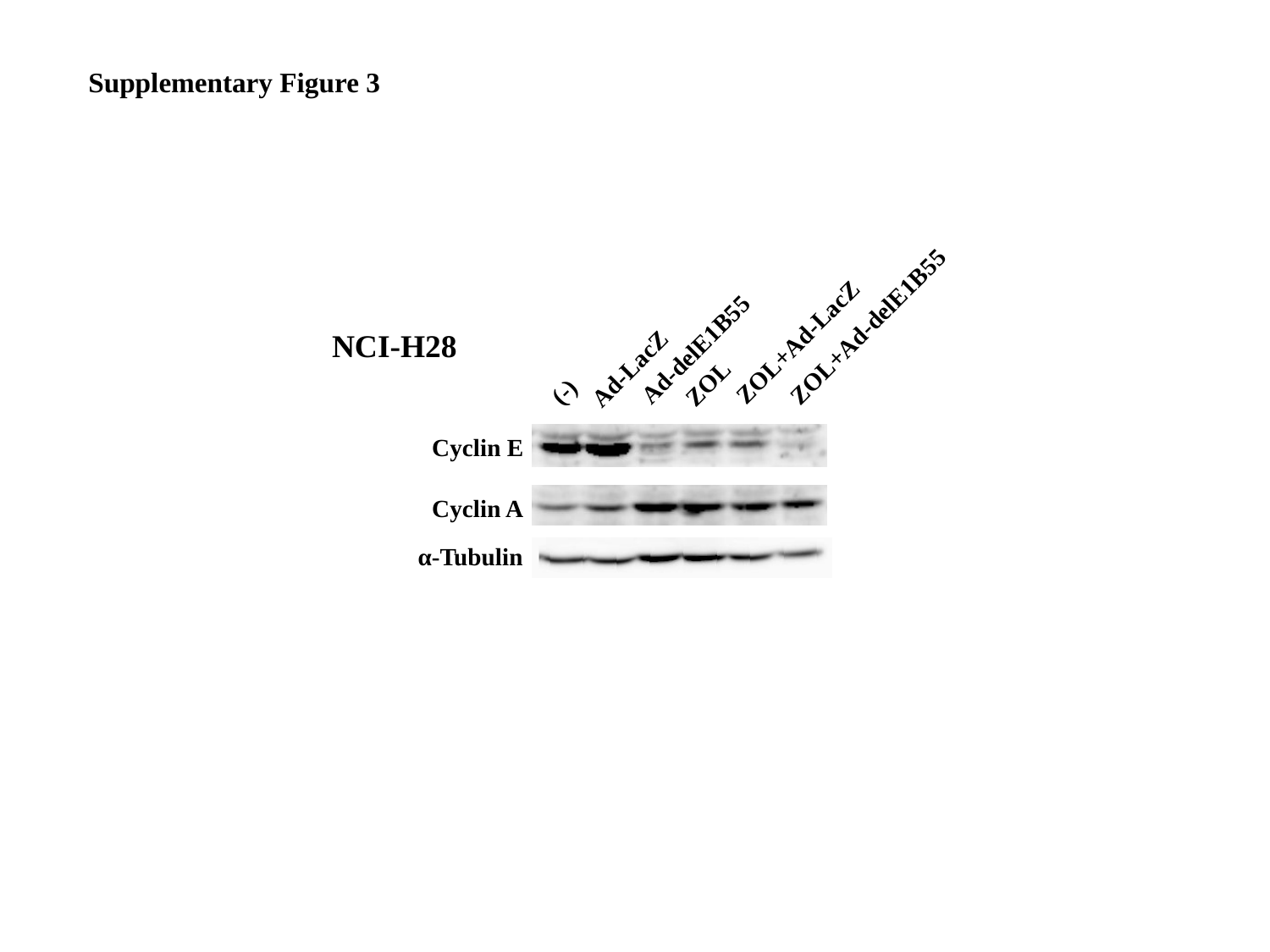

Supplementary Figure 3
ZOL+Ad-delE1B55
ZOL
ZOL+Ad-LacZ
NCI-H28
Ad-delE1B55
Ad-LacZ
(-)
Cyclin E
Cyclin A
α-Tubulin
