# Supplementary material for: Combination of a third generation bisphosphonate and replication-competent adenoviruses augments the cytotoxicity on mesothelioma
Source: BMC Cancer. 2016 Jul 12;16:455. doi: 10.1186/s12885-016-2483-y (PMC4942884; doi:10.1186/s12885-016-2483-y)
Supplement: Additional file 4: Table S1. — Expression Ad receptor molecules after ZOL treatments on NCI-H28 cells. (DOCX 21 kb) [file 12885_2016_2483_MOESM4_ESM.docx]

**Supplementary Table 1. Expression Ad receptor molecules after ZOL treatments on NCI-H28 cells.**

| **Ab** | **ZOL**  **treatment** | **Mean fluorescent intensity**  **(average±SE)** | **% radio**  **(average±SE)** |
| --- | --- | --- | --- |
| 2nd Ab alone | (-) | 11.39±0.03 |  |
|  | 10 μM | 12.29±0.03 | 107.90±0.21 |
|  | 40 μM | 12.80±0.06 | 112.38±0.50 |
|  | 80 μM | 11.55±0.86 | 101.38±0.79 |
| CAR | (-) | 127.26±0.31 |  |
|  | 10 μM | 128.06±0.39 | 100.63±0.39 |
|  | 40 μM | 125.02±0.44 | 98.24±2.78 |
|  | 80 μM | 119.29±0.69 | 93.73±0.03 |
| Integrin αvβ3 | (-) | 1006.61±1.24 |  |
|  | 10 μM | 985.55±2.04 | 97.91±0.37 |
|  | 40 μM | 984.40±0.06 | 97.79±0.04 |
|  | 80 μM | 718.29±2.33 | 71.36±0.12 |
| Integrin αvβ5 | (-) | 44.01±0.09 |  |
|  | 10 μM | 30.58±0.06 | 69.48±0.20 |
|  | 40 μM | 30.67±0.04 | 69.70±0.14 |
|  | 80 μM | 22.01±0.03 | 50.01±0.13 |

NCI-H28 cells untreated or treated with ZOL at the indicated concentrations for 48 hrs were subjected to flow cytometry to detect expression levels of the cellular receptors for type 5 Ad. Means fluorescence intensity was expressed as an arbitrary unit and influence of ZOL on the expression levels was expressed as a percent ratio based on respective ZOL-untreated cases as a control (n=3). Data of ZOL at 80 μM are the same as those in Table 3.
