# Supplementary material for: Combination of a third generation bisphosphonate and replication-competent adenoviruses augments the cytotoxicity on mesothelioma
Source: BMC Cancer. 2016 Jul 12;16:455. doi: 10.1186/s12885-016-2483-y (PMC4942884; doi:10.1186/s12885-016-2483-y)
Supplement: Additional file 5: Figure S4. — Influence of ZOL on Ad infectivity. NCI-H28 cells were infected with Ad-GFP (300 or 1,000 vp/cell) and were treated with ZOL as indicated for 48 h. The mean fluorescent intensity of the GFP-positive cells was analyzed with flow cytometry and expressed with an arbitrary unit. Averages and the SE bars are shown (n = 3). Data of ZOL at 80 μM are the same as those in Fig. 6b. (PPTX 46 kb) [file 12885_2016_2483_MOESM5_ESM.pptx]

## Slide 1
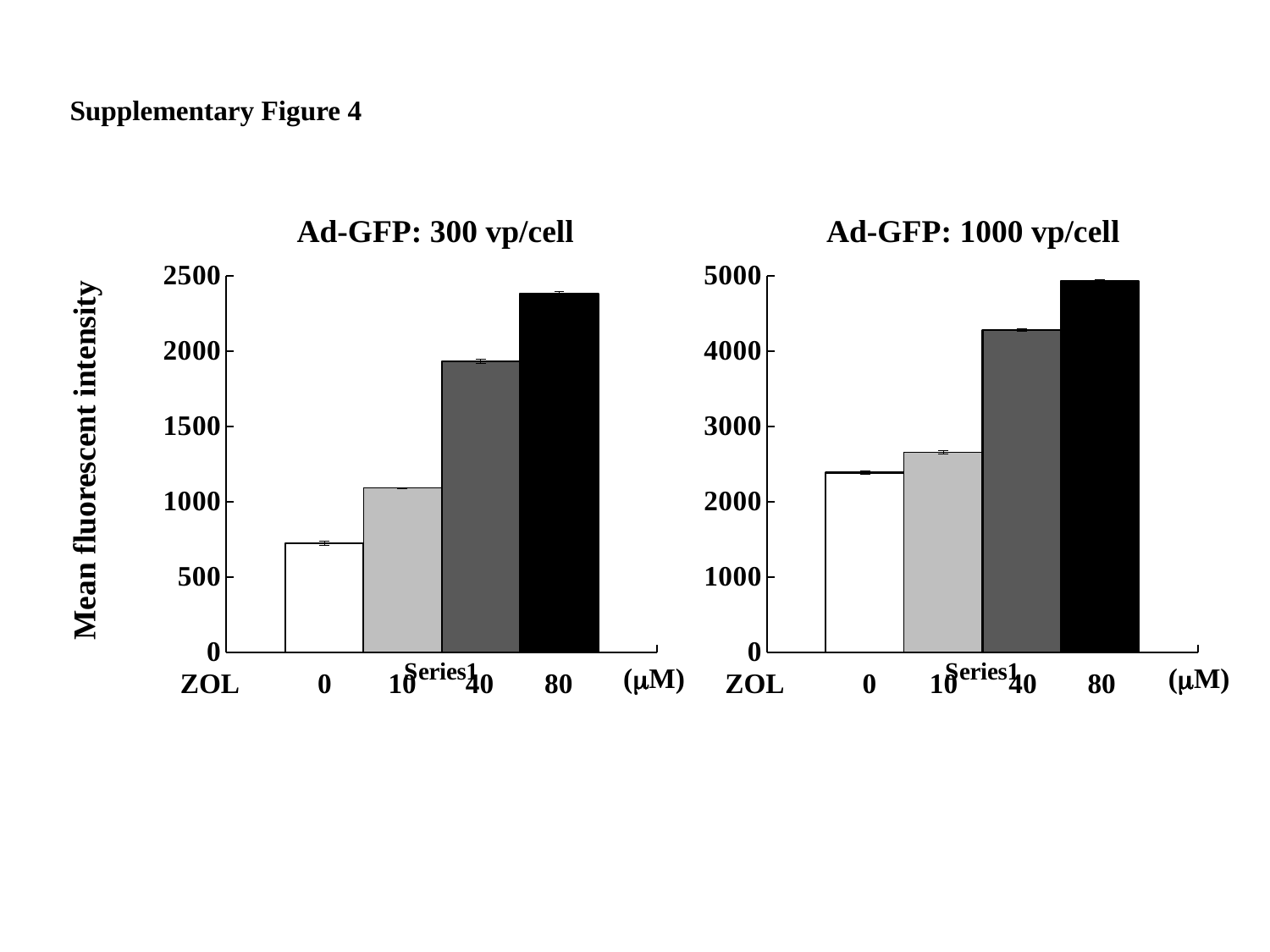

Supplementary Figure 4
Ad-GFP: 300 vp/cell
Ad-GFP: 1000 vp/cell
### Chart
| Category | Ad MOI=300 | 10 uM | 40 uM | 80 uM |
|---|---|---|---|---|
| | 724.62 | 1089.063333333333 | 1930.36 | 2380.633333333334 |
### Chart
| Category | Ad MOI=300 | 10 uM | 40 uM | 80 uM |
|---|---|---|---|---|
| | 2385.34 | 2654.836666666667 | 4278.046666666667 | 4927.94 |Mean fluorescent intensity
(mM)
ZOL
0
10
40
80
(mM)
ZOL
0
10
40
80
